# Neuron‐specific deletion of CuZnSOD leads to an advanced sarcopenic phenotype in older mice

**DOI:** 10.1111/acel.13225

**Published:** 2020-09-04

**Authors:** Shylesh Bhaskaran, Natalie Pollock, Peter C. Macpherson, Bumsoo Ahn, Katarzyna M. Piekarz, Caroline A. Staunton, Jacob L. Brown, Rizwan Qaisar, Aphrodite Vasilaki, Arlan Richardson, Anne McArdle, Malcolm J. Jackson, Susan V. Brooks, Holly Van Remmen

**Affiliations:** ^1^ Aging & Metabolism Research Program Oklahoma Medical Research Foundation Oklahoma City OK USA; ^2^ Department of Musculoskeletal Biology Institute of Ageing and Chronic Disease MRC‐Arthritis Research UK Centre for Integrated Research into Musculoskeletal Ageing (CIMA) University of Liverpool Liverpool UK; ^3^ Department of Molecular and Integrative Physiology University of Michigan Ann Arbor MI USA; ^4^ Oklahoma Center For Neuroscience University of Oklahoma Health Sciences Center Oklahoma City OK USA; ^5^ Oklahoma City VA Medical Center Oklahoma City OK USA; ^6^ Department of Biochemistry and Molecular Biology University of Oklahoma Health Sciences Center Oklahoma City OK USA

**Keywords:** aging, CuZnSOD, denervation, motor neuron, sarcopenia

## Abstract

Age‐associated loss of muscle mass and function (sarcopenia) has a profound effect on the quality of life in the elderly. Our previous studies show that CuZnSOD deletion in mice (*Sod1* ^−/−^ mice) recapitulates sarcopenia phenotypes, including elevated oxidative stress and accelerated muscle atrophy, weakness, and disruption of neuromuscular junctions (NMJs). To determine whether deletion of *Sod1* initiated in neurons in adult mice is sufficient to induce muscle atrophy, we treated young (2‐ to 4‐month‐old) Sod1flox/SlickHCre mice with tamoxifen to generate *i*‐mn‐Sod1KO mice. CuZnSOD protein was 40‐50% lower in neuronal tissue in *i*‐mn‐Sod1KO mice. Motor neuron number in ventral spinal cord was reduced 28% at 10 months and more than 50% in 18‐ to 22‐month‐old *i*‐mn‐Sod1KO mice. By 24 months, 22% of NMJs in *i‐*mn‐Sod1KO mice displayed a complete lack of innervation and deficits in specific force that are partially reversed by direct muscle stimulation, supporting the loss of NMJ structure and function. Muscle mass was significantly reduced by 16 months of age and further decreased at 24 months of age. Overall, our findings show that neuronal‐specific deletion of CuZnSOD is sufficient to cause motor neuron loss in young mice, but that NMJ disruption, muscle atrophy, and weakness are not evident until past middle age. These results suggest that loss of innervation is critical but may not be sufficient until the muscle reaches a threshold beyond which it cannot compensate for neuronal loss or rescue additional fibers past the maximum size of the motor unit.

## Graphical abstract

Using deletion of CuZnSOD in motor neurons to induce increased oxidative stress and mimic loss of motor neurons in spinal cord, we show that neuronal loss induces NMJ disruption that progresses over time to cause muscle atrophy and weakness in *i*‐mn‐Sod1KO mice. The delay between loss of motor neuron number and significant atrophy and weakness suggests there are compensatory mechanisms at play to mitigate the impact of reduced innervation (possibly including increased sprouting) that eventually fail over time.

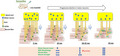

## 1 INTRODUCTION

Age‐associated loss of skeletal muscle mass and function, or sarcopenia, is a major determinant of reduced quality of life and frailty in the elderly. While the pathogenesis of sarcopenia is multifactorial, a progressive increase in age‐associated oxidative stress is considered a major contributor to muscle impairment in elderly humans (Barker & Traber, 2007) as well as in experimental rodent models (Broome et al., 2006; Vasilaki et al., 2007). In a series of previous studies, we and others have reported that mice lacking the superoxide scavenger Copper Zinc Superoxide Dismutase (*Sod1* ^−/−^ mice) show a very high level of oxidative stress in all tissues, associated with a number of phenotypes associated with aging including hearing loss, cataracts, skin thinning, delayed wound healing, and an accelerated sarcopenia phenotype (Deepa et al., 2019; Muller et al., 2006; Snider, Richardson, Stoner, & Deepa, 2018). In particular, hind limb skeletal muscle in *Sod1* ^−/−^ mice exhibits mitochondrial abnormalities, degeneration of neuromuscular junctions (NMJs) and loss of innervation, and loss of contractile force during early adulthood that is similar to the hind limb muscle phenotype in old wild‐type mice (Brooks & Faulkner, 1991, 1994; Jang et al., 2010; Larkin et al., 2011). The clear changes in motor neuron and neuromuscular junction morphology point to a coordinated role of motor neurons and skeletal muscle in the development of sarcopenia.

To delineate the relative roles of pre‐ and postsynaptic redox imbalance in sarcopenia, we previously generated mice with a genetic deletion of *Sod1* specifically targeted to skeletal muscle and surprisingly found that CuZnSOD deletion targeted to skeletal muscle does not induce loss of muscle mass even in older adult mice (up to 16–17 months) and contractile dysfunction is only marginally reduced (Zhang et al., 2013). This result suggests that the muscle is not the sole effector of the sarcopenia phenotype. Conversely, to determine whether the muscle decline associated with aging is initiated by redox changes in motor neurons, we generated a mouse model in which the human CuZnSOD gene was specifically targeted to neurons on an *Sod1* ^−/−^ background (*SynTgSod1* ^−/−^ mice) (Sakellariou et al., 2014). In these “rescue” mice, the neuron‐specific expression of *Sod1* prevented the muscle atrophy, NMJ degeneration, and muscle weakness phenotypes that occur in the *Sod1* ^−/−^ mice suggesting that the redox balance in motor neuron is critical mediator of muscle innervation and atrophy (Sakellariou et al., 2014). In support of this, ablation of the antioxidant enzyme glutathione peroxidase‐4 specifically in motor neurons also causes muscle atrophy and paralysis in mice (Chen, Hambright, Na, & Ran, 2015). Thus, we hypothesized that reduction of *Sod1* in motor neurons would be sufficient to induce muscle impairment in wild‐type mice.

To test this hypothesis, we previously generated mice with a constitutive embryonic neuron‐specific deletion of *Sod1* using the nestin‐cre (*nSod1* ^−/−^ mice) promoter (Sataranatarajan et al., 2015). Surprisingly these mice did not show significant atrophy of gastrocnemius muscles even at 20 months of age, although mild contractile dysfunction and signs of denervation were evident. We hypothesized that the deletion of Sod1 using the nestin‐cre was not sufficient to induce a phenotype or that the deletion during embryonic development may induce compensatory effects that altered the effect of motor neuron deletion in the mice at later ages. To address these possibilities and to measure the effect of *Sod1* deficiency in motor neurons on muscle mass and function induced in adult mice, we generated a mouse model with inducible deletion of *Sod1* (*i*‐mn‐Sod1KO) targeted by a neuron‐specific cre recombinase driven by the Thy1 promoter. The findings presented here show that neuron deletion of *Sod1* in adult mice does indeed result in atrophy and contractile dysfunction of the gastrocnemius muscle as well as a disruption of NMJ morphology in older mice. Together these data support a critical role of motor neurons in initiating muscle atrophy, but also suggest it is a progressive effect that requires time, intrinsic effects of aging on tissue function to develop, or an exhaustion of the ability of neurons to compensate for neuronal deficits.

## 2 MATERIALS AND METHODS

### 2.1 Generation of motor neuron deficient *Sod1* mice (*i*‐mn‐Sod1KO)

The SlickH Cre mice were obtained from Jackson Laboratories (Bar Harbor, ME Stock # 012708). These mice have previously been shown to induce expression of Cre recombinase in motor neurons in the spinal cord without expression in non‐neuronal tissues, allowing us to generate a motor neuron deletion without any effect on muscle *Sod1* (Young et al., 2008). Sod1^flox/flox^ mice were generated in our laboratory and have previously been described (Zhang et al., 2013). Both strains are maintained on a C57Bl6 background. The *i*‐mn‐Sod1KO mice were generated by two‐step cross‐breeding between Sod1^flox/flox^ and SlickH Cre mice. Sod1^floxflox;SlickHCre^ (*i*‐mn‐Sod1KO) and littermate Sod1^flox/flox; SlickHwt^ controls were used for the study. Tamoxifen was dissolved in corn oil (10 mg/ml) and administered to 2‐ to 4‐month‐old Sod1^floxflox;SlickHCre^ (*i*‐mn‐Sod1KO) and littermate Sod1^flox/flox; SlickHwt^ controls by intraperitoneal injection for two rounds of 5 consecutive days (60 mg/kg body weight). Mice were sacrificed at various ages as noted in each particular dataset. Previously, we found that whole‐body deletion of *Sod1* in mice (*Sod1* ^−/−^) results in a lower body weight (15‐20%) and accelerated loss of muscle mass with age that mimics the sarcopenia phenotype (Muller et al., 2006; Sakellariou et al., 2011). In *i*‐mn‐Sod1^−/−^ mice, we did not see any change in body weight compared with age‐matched tamoxifen‐treated control mice (data not shown).

### 2.2 Western blotting

Western blot analysis was performed as previously described (Bhaskaran et al., 2018). Protein bands were detected by ECL reagent using G‐Box (Syngene), and the band intensity was measured by NIH image J. The following primary antibodies were used: CuZnSOD (Enzo), β‐actin (Sigma) VDAC (Cell Signaling), MURF1 (Santa Cruz).

### 2.3 Immunofluorescence

Motor neuron count and area in lumbar region of spinal cord were determined as described previously (Piekarz et al., 2020). Sections were stained with NeuN antibody (Cell Signaling Technology) followed by Alexa Fluor^®^488 F(ab)2 Fragment of Goat Anti‐Rabbit IgG (H + L) antibody. Images were taken with Nikon Eclipse confocal microscope and analyzed with Nikon elements c image acquisition software. NeuN is a protein that is present in nucleus and perinuclear cytoplasm of neurons and is widely used as neuronal marker (Gusel'nikova & Korzhevskiy, 2015).

### 2.4 Muscle function analyses

Gastrocnemius (GTN) muscle contractile properties were measured *in situ* as previously described by (Larkin et al., 2011). Briefly, whole GTN muscle was isolated in anesthetized mice, and the distal tendon was severed and secured to the lever arm of a servomotor (model 305B; Aurora Scientific Inc.). The muscle was activated by stimulation of the tibial nerve using a bipolar platinum wire electrode. The voltage of single 0.2 ms stimulation pulses and subsequently muscle length were adjusted to give a maximum isometric twitch. With the muscle held at optimal length for maximum twitch force (*L*_o_), 300 ms trains of pulses were applied at increasing stimulation frequencies until the maximum isometric tetanic force (*P*_o_) was achieved. Subsequently, a cuff electrode was placed around the proximal and distal ends of the muscle for direct muscle stimulation, and the *P*_o_ was once again determined. GTN muscle fiber length (*L*_f_) was calculated by multiplying *L*_o_ by 0.45. Total fiber cross‐sectional area (CSA) was calculated by dividing the muscle mass (mg) by the product of *L*_f_ (mm) and the density of mammalian skeletal muscle, 1.06 g/cm^2^. Specific *P*_o_ (N/cm^2^) was calculated by dividing *P*_o_ by total fiber CSA for each muscle (Brooks & Faulkner, 1988).

### 2.5 Immunofluorescence analysis of NMJS

Small fiber bundles (10–20 muscle fibers) or 35 mm thick longitudinal sections from GTN muscles were prepared for NMJ analysis as described previously (Macpherson, Farshi, & Goldman, 2015). Briefly, muscles were fixed for 15 min at room temperature in 4% paraformaldehyde, rinsed with PBS, cryoprotected with a gradient of 30% sucrose, frozen in OTC (Triangle Biomedical Sciences) and cut into longitudinal sections. Bundles of muscle fibers were teased from some of the remaining muscle blocks after being thawed in PBS. Muscle sections and fiber bundles were blocked for 30 min in PBS containing 5% donkey serum and 1% triton X‐100. To visualize axons and presynaptic terminals, muscles were incubated overnight at 4°C with rabbit anti‐βIII‐tubulin antibody (1:1000; BioLegend, 802001) and/or mouse anti‐SV2 antibody (1:50; Developmental Studies Hybridoma Bank, SV2). After rinsing in PBS a minimum of three times to remove excess primary antibodies, muscles were incubated with Alexa Fluor 594‐conjugated α‐bungarotoxin (α‐Btx; 1:2000; Thermo Fisher, B‐13423) to label acetylcholine receptors on the postsynaptic muscle membrane. Following incubation with a combination of rabbit and mouse Alexa 488 secondary antibodies (1:2000 Thermo Fisher, A11034 and A21202), slides were washed, coverslips applied and the slides were examined with either an Olympus FV1000 or Nikon A1 laser scanning confocal microscope. Quantitative analysis of NMJ structure was done by two different individuals using software associated with the Olympus FV1000 microscope or with ImageJ. At least 4 muscles were analyzed for each group and approximately 100 endplates were scored in each muscle. Depending on the extent of overlap of βIII‐tubulin/SV2 with α‐Btx, endplates were scored as completely innervated (100% overlap), partially denervated (∼10%–80% overlap) or completely denervated (Jang et al., 2010; Macpherson et al., 2015). Differences for each classification of innervation status between experimental groups were established by analysis of variance (ANOVA) with individual differences determined by Tukey's post hoc tests.

### 2.6 Simultaneous high‐resolution respirometry and fluorometry measurements

Skeletal muscle fibers were permeabilized as previously described (Ahn et al., 2019) and used for simultaneous measurement of oxygen consumption rate (OCR) and mitochondrial hydrogen peroxide production using the Oxygraph‐2k (O2k, OROBOROS Instruments). Data for both OCR and rates of H_2_O_2_ generation were normalized by milligrams of muscle bundle wet weights.

### 2.7 Measurement of F2‐isoprostanes and protein carbonyls in skeletal muscle tissue

F_2_ isoprostanes isolated from 150 mg of tissues were measured using gas‐chromatography coupled with mass spectrophotometry [GC‐MS] as described previously (Ahn et al., 2019). The level of F_2_‐isoprostanes in muscle tissues was calculated based on the internal standard, and the amount was expressed as nanograms of 8‐Iso‐PGF2α, per gram of muscle tissue. Protein carbonylation in gastrocnemius muscle was determined as we have previously described, using 25 mg of frozen tissue (Qaisar et al., 2018). The carbonyl content of the protein samples was expressed as the ratio of FTC fluorescence (carbonyls) to Coomassie blue absorption (protein concentration).

### 2.8 Quantitative real‐time PCR

Total RNA was extracted using the Trizol) from 30 mg of frozen GTN muscle as described before (Sataranatarajan et al., 2015). Primers used are shown in Table 1.

**TABLE 1:** List of QPCR primers

Gene	Primer sequence
Atrogin‐1	
F	AACCGGGAGGCCAGCTAAAGAACA
R	TGGGCCTACAGAACAGACAGTGC
AChR‐α	
F	ACCTGGACCTATGACGGCTCT
R	AGTTACTCAGGTCGGGCTGGT
ATPase6	
F	CACAACACTAAAGGACGAACCT
R	GGGATGGCCATGGCTAGGTTTA
COX2	
F	ATGGCCTACCCATTCCAACT
R	CGGGGTTGTTGATTTCGTC
Rieske	
F	TGGTCTCCCAGTTTGTTTCC
R	GCAGCTTCCTGGTCAATCTC
SDHA	
F	CAGAAGTCGATGCAGAACCA
R	CGACCCGCACTTTGTAATCT
NDUF3	
F	CTGTGGCAGCACGTAAGAAG
R	ACTCATCAAGGCAGGACACC
18SRNA	
F	GTGGAGCGATTTGTCTGGTT
R	CCC ACT GTG TGC ATT CCA GAT TGG
MURF1	
F	GAGAACCTGGAGAAGCAGCT
R	CCGCGGTTGGTCCAGTAG
LC3	
F	TTGGTCAAGATCATCCGGC
R	GCTCACCATGCTGTGCTGG
p62	
F	AGGCGCACTACCGCGAT
R	CGTCACTGGAAAAGGCAACC
Bnip3	
F	GCTCCCAGACACCACAAGAT
R	TGAGAGTAGCTGTGCGCTTC

## 3 RESULTS

### 3.1 Motor neuron deletion of the *Sod1* gene reduces CuZnSOD protein expression in motor neurons

**FIGURE 1:**
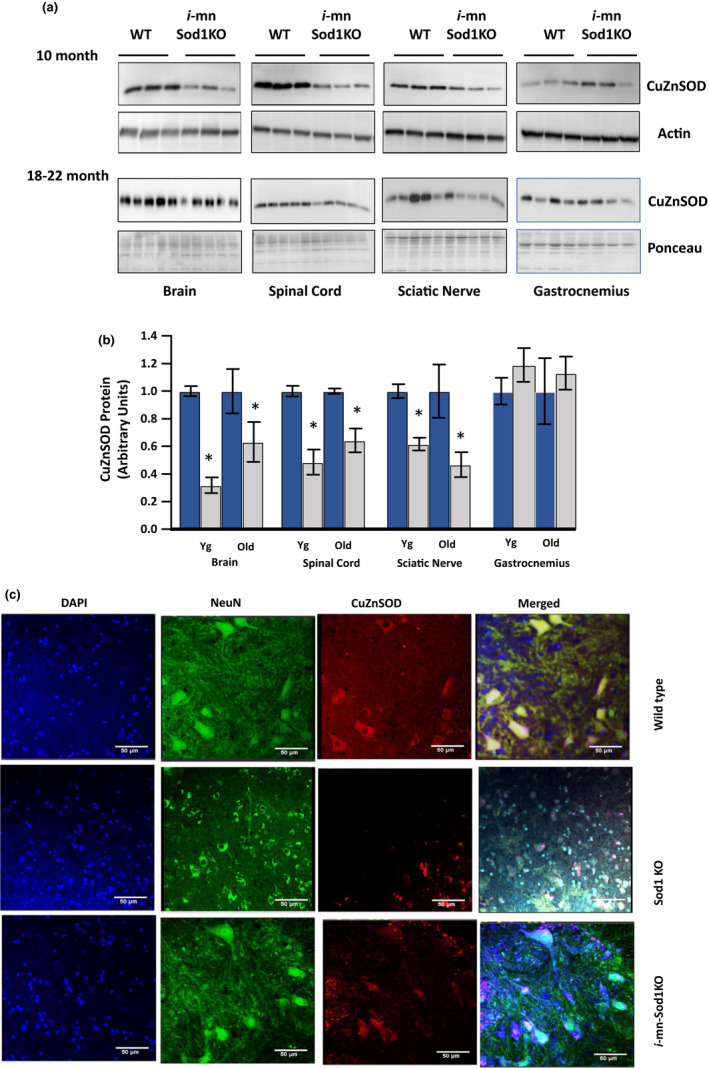
Characterization of the *i*‐mn‐Sod1KO model. (a) Representative Western blot analysis of CuZnSOD expression in neuronal and non‐neuronal tissues (brain, spinal cord, sciatic nerve, and gastrocnemius muscle) in 10‐month‐old and 18‐ to 22‐month‐old wild‐type and *i*‐*mn*‐*Sod1 KO* mice (*n* = 3–5 per group, mixed male and female). Mice were injected with tamoxifen at 2–4 months of age. (b) Graphical representation of quantified blots, normalized to β‐actin or Ponceau stain. Bars represent mean ± *SEM* (*n* = 3–5 per group) and are expressed relative to wild‐type control value for each age group. Student's unpaired *t* test, *p ≤ .05. (c) Representative image of immunofluorescence staining of spinal cord lumbar region from control (10‐month‐old, top panel), Sod1KO (10‐month‐old, middle panel) and *i*‐mn‐Sod1KO mice (10‐month‐old, bottom panel) with DAPI (blue, first panel), NeuN (green, second panel), CuZnSOD (red, third panel). A merged image of DAPI, NeuN, and CuZnSOD is given in panel 4. Scale 50 µm, magnification 20×

The SlickH Cre mouse was originally developed as a model to delete genes in motor neurons, while also simultaneously labeling the neurons with YFP. When crossed to mice carrying the Sod1 floxed allele that we had previously generated (Zhang et al., 2013), this Cre mouse model allows us to achieve deletion of the mouse *Sod1* gene under tamoxifen induction of Cre recombinase targeted by the Thy1 promoter to all motor neurons, along with motor neuron targeted expression of YFP in the same cells (*i*‐mn‐Sod1KO mice). It should be noted that while we have targeted motor neurons with this model, the expression is not restricted to only motor neurons. Figure 1 (panels a and b) shows the effect of tamoxifen induction of *Sod1* deletion initiated in 2‐ to 4‐month‐old mice on CuZnSOD expression in neuronal tissue (brain, spinal cord, and sciatic nerve) and non‐neuronal (GTN muscle) tissues from young (10 months) and old (18‐22 months) control (*Sod1* ^floxflox;SlickHwt^) and *i*‐mn‐Sod1KO (*Sod1* ^floxflox;SlickHCre^) mice. At 10 months of age (6‐8 months post‐tamoxifen), CuZnSOD protein expression is significantly reduced in brain (~68%), spinal cord (~51%), and sciatic nerve (~41%) in *i*‐mn‐Sod1KO mice, compared with age‐matched wild‐type control mice, while CuZnSOD protein expression in skeletal muscle, spleen (Figure S4), and liver (data not shown) is unchanged. At 18–20 months of age, CuZnSOD protein expression remains reduced in brain (37%), spinal cord (~36%), and sciatic nerve (~53%) in *i*‐mn‐Sod1KO mice compared with age‐matched control mice while the expression in GTN muscle is not reduced. It should be noted that the Western blot analysis is from tissue homogenates that are composed of multiple cell types, some of which will not express the Cre recombinase and thus will not display deletion of *Sod1*. Thus, we expect to see only a partial reduction by Western blot even though the actual reduction in the motor neurons is predicted to be more complete. The reduction of *Sod1* in neuronal tissues does not result in a compensatory increase in the protein level of the mitochondrial form of superoxide dismutase (MnSOD) (data not shown). To further confirm the impact of the deletion of *Sod1* in *i*‐mn‐Sod1KO mice, we sectioned the lumbar spinal cord from control, *i*‐mn‐Sod1KO, and *Sod1* ^−/−^ mice and stained with antibodies specific for CuZnSOD and NeuN. As shown in Figure 1c, control mice show clear co‐localization of CuZnSOD and NeuN in the spinal cord that is absent in sections from the *i*‐mn‐Sod1KO or in *Sod1* ^−/−^ mice, supporting the deletion of *Sod1* in motor neurons in the *i*‐mn‐Sod1KO mouse model. The Slick H Cre model is also expressed in sensory neurons in the spinal cord but we would not expect that to alter the muscle phenotype we are investigating in this study. Motor neurons in the cortex are also targeted for *Sod1* deletion as indicated by the reduction in CuZnSOD expression in brain. The potential impact of cortical deletion of *Sod1*, while potentially interesting, is beyond the scope of our study.

### 3.2 Motor neuron ablation of Sod1 reduces the number and area of alpha motor neurons

**FIGURE 2:**
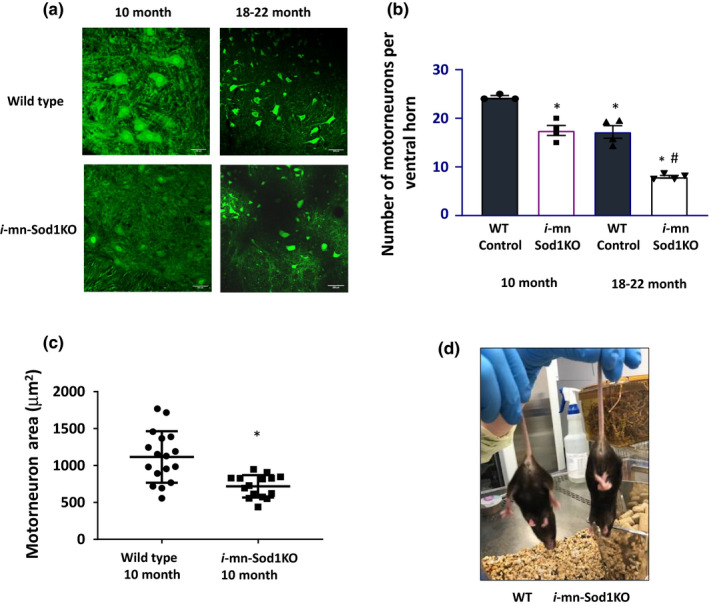
Reduced motor neuron number and motor coordination in *i*‐*mn*‐*Sod1 KO* mice. (a) Representative image showing NeuN immunostaining (green) in young (10 months) and old (18–22 months) wild‐type control (top panel) and *i*‐mn‐Sod1‐KO mice (bottom panel). A transverse section of lumbar region of spinal cord was used for staining. Scale 200 µm, magnification 20×. (b) Quantification of motor neuron numbers from the lumbar region of spinal cord of young (10 months) and old (18–22 months) in control and *i*‐mn‐Sod1KO mice (*n* = 3–4 mice/group). *Significantly different from 10‐month control and (#) from 18‐ to 22‐month control by one‐way ANOVA (*p* < .05) (c) Quantification of motor neuron area from young (10 month) control and *i*‐mn‐Sod1KO mice (*n* = 4 mice/group) *Significantly different from control by Student's *t* test (*p* < .05). (d) Loss of hind limb extension reflex *i*‐mn‐Sod1KO mice. Representative pictures illustrating the hind limb clasping reflex in 22‐month‐old control (left) and *i*‐mn‐Sod1KO mice (right)

As shown in Figure 2a,b, the number of alpha motor neurons present in the lumbar spinal cord from 18‐ to 22‐month‐old control mice as measured by NeuN staining is reduced by 30% compared with 10‐month‐old control mice. The loss of alpha motor neurons is greater in *i*‐mn‐*Sod1* ^−/−^ mice, reaching ~50% fewer motor neurons in old *i*‐mn‐*Sod1* ^−/−^ mice compared with old control mice. In addition to a loss in motor neuron number, the area of the motor neurons in the older *i*‐mn‐*Sod1* ^−/−^ mice is approximately 40% smaller compared with age‐matched wild‐type mice as shown in Figure 2c. These findings suggest the preferential loss of larger motor neurons in the *i*‐mn‐*Sod1* ^−/−^ mice. By age 24 months, the *i*‐mn‐*Sod1* ^−/−^ mice also show dramatic clasping behavior when suspended by the tail, a classic indicator of motor neuron degeneration (Figure 2d). It is possible that this phenotype is also partially induced by loss of *Sod1* in proprioceptive sensory neurons in the *i*‐mn‐Sod1KO mice.

### 3.3 *i*‐mn‐Sod1KO mice show an increase in neuromuscular junction degeneration that occurs later in life

**FIGURE 3:**
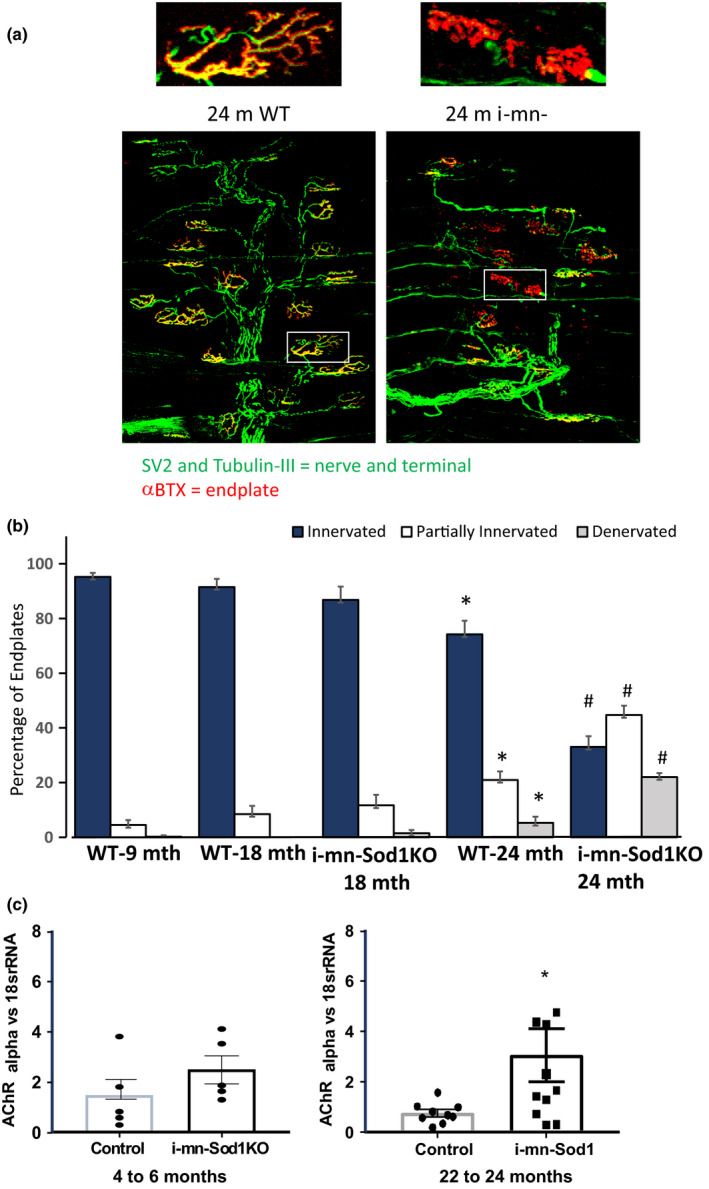
Neuromuscular junction (NMJ) morphology. (a) Representative images of NMJs in gastrocnemius muscles of 24‐month‐old WT and *i*‐mn‐Sod1KO mice. Expanded insets at top correspond to respective boxed NMJs below and display some of the more dramatic differences observed between 24‐month‐old WT and *i*‐mn‐Sod1KO. Note the decreased complexity, lack of innervation, and increased endplate fragmentation in *i*‐mn‐Sod1KO. (b) Quantification of NMJs in which pre‐ and postsynaptic structures show full overlap (Innervated), partial overlap (Partially Innervated), and no overlap (Denervated). Four muscles per group were analyzed and approximately 100 endplates were evaluated for each muscle. Data are presented as means ± *SD*. Differences between experimental groups for each state if innervation is shown with * indicating differences from all other groups and # also different from all other groups, that is, a significant increase in denervated NMJs is observed by 24 months in WT mice that was more severe in i‐mn‐Sod1KO mice. (c) Quantification of mRNA for AchR alpha relative to 18S RNA using real‐time PCR analysis. Values represent mean ± *SEM*. *Different from wild‐type control by Student's *t* test (*p* < .05). *n* = 9–11 (male and female)

We have previously reported a dramatic increase in denervated neuromuscular junction (NMJs) and fragmentation of acetylcholine receptors in hind limb muscle from the *Sod1* ^−/−^ mice that is present even in young adult *Sod1* ^−/−^ mice (Jang et al., 2010). We hypothesized that motor neuron deletion of CuZnSOD expression would alter motor neuron function and that would be sufficient to cause the disruption of NMJ structure and function. To test this hypothesis, we examined the NMJ structure of the GTN muscle using immunofluorescent staining techniques (Figure 3a) in *i*‐mn‐Sod1KO and control mice throughout the lifespan. Figure 3b shows the results of quantitative analysis of the extent of overlap between pre‐ and postsynaptic structures. In control mice, nearly 100% of the NMJs show complete overlap between nerve terminals and motor endplates out to 18 months. By 24 months, close to 20% of NMJs in control muscles are partially innervated and 5% are fully denervated. In contrast, in muscles of i‐mn‐Sod1KO mice, ~10% of NMJs were partially denervated by 18 months, and by 24 months this number had increased to 45% with only 33% ±4% (SD) of the NMJs remaining fully innervated. It is important to note that even at 24 months of age the *i*‐mn‐Sod1KO mice only have 22% of the endplates fully denervated. Consistent with the histological evidence of denervation we observed a modest but significant increase in transcript levels of the alpha subunit of nAChR (Figure 3c).

### 3.4 Age‐associated skeletal muscle atrophy is accelerated in *i*‐mn‐Sod1KO mice

**FIGURE 4:**
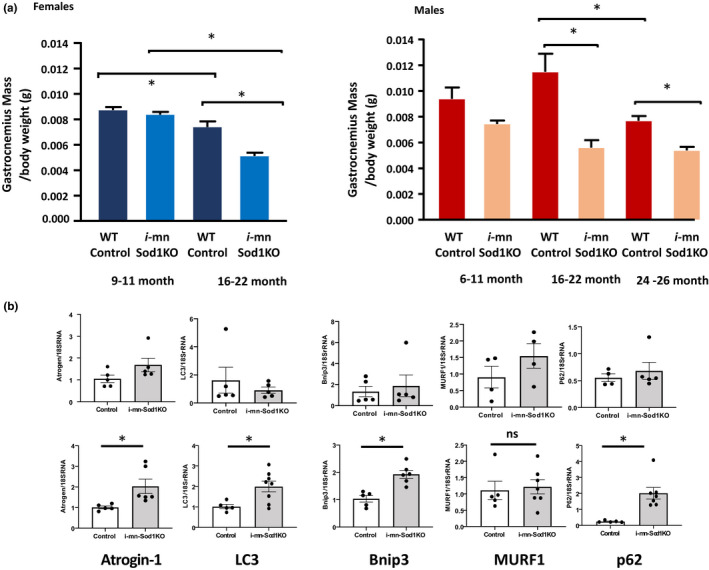
Analysis of gastrocnemius muscle mass in male and female wild‐type and *i*‐mn‐Sod1KO mice as a function of age. (a) Gastrocnemius muscle mass relative to body mass at 6–11‐months (*n* = 23 female WT; 4 male WT; 39 *i*‐mn‐Sod1KO females; 6 *i*‐mn‐SodKO males), 16–22‐months (*n* = 16 female WT; 10 male WT; 37 *i*‐mn‐Sod1KO females; 14 *i*‐mn‐SodKO males) and in 24‐ to 26‐month‐old males (*n* = 21 WT and 21 *i*‐mn‐Sod1KO mice). *Difference between marked groups detected using one way ANOVA (*p* < .05) Data represent mean ± *SEM* for each group. Females are shown in blue and males in red. (b) Quantification of mRNA expression in gastrocnemius muscle tissue from 24‐month‐old control and i‐mn‐Sod1 KO mice (*n* = 5–8) for Atrogin‐1, MURF1, LC3, BNIP3, and P62 (b)

Figure 4 shows the effect of CuZnSOD deletion on GTN muscle mass. Muscle mass is not different between control and *i*‐mn‐Sod1KO mice up to 11 months of age in males or females. There is a 24% loss in GTN muscle mass relative to body mass in older (16–22 months) compared with young adult (9–11 months) female control mice and an additional 30% loss in muscles from 16‐ to 22‐month‐old *i*‐mn‐Sod1KO mice compared with age‐matched female controls. For comparison, in a previous study we reported a 42% difference in GTN muscle mass relative to body mass in 20‐month‐old female wild‐type and *Sod1* ^−/−^ mice (Muller et al., 2006). In male mice, GTN muscle mass is 18% lower in 22‐ to 26‐month‐old versus 6‐ to 11‐month‐old control mice and there is a 51% loss in *i*‐mn‐Sod1KO male mice versus control at 16‐22 months and 30% difference between these groups at 24–26 months.

In addition to the accelerated onset and severity of atrophy in GTN muscles, masses of EDL, soleus, TA, and Quad muscles show an acceleration of age‐associated atrophy. No differences were observed between male and female mice for muscle mass normalized for body mass at any age for either strain of mice. Thus, data from males and females were pooled and are shown in Figure S1. These data show that for control mice, muscle mass is not different between 11 months and 16 months for EDL, soleus, TA or Quad muscles, but generally declines by 24 months of age. In contrast, while muscle mass is not different between control and *i*‐mn‐Sod1KO mice at 11 months of age, muscle masses for *i*‐mn‐Sod1KO mice decline by 16 months in all cases. Furthermore, for both TA and Quad muscles a steady decline in mass occurs throughout all of the ages studied. These findings indicate that the reduction in CuZnSOD levels in motor neurons can impact a number of hind limb muscles tending to accelerate the reduction in muscle mass that occurs with age.

To probe the molecular changes associated with loss of muscle mass, we measured the expression of several genes associated with protein breakdown as shown in Figure 4c. We measured a significant increase in atrogin‐1 (2.5‐fold) in 24‐month‐old *i*‐mn‐Sod1KO mice compared to control mice, with no change in the expression of MURF1 (Figure 4c). Despite the lack of change in gene expression in muscle from older i‐mn‐Sod1KO mice, the protein expression of MURF1 was significantly upregulated (2.4‐fold) in *i*‐mn‐Sod1KO mice muscle (Figure S2). Analysis of transcript levels of markers of autophagy showed a significant increase in LC3 (1.9‐fold), p62 (2‐fold), and BNIP3 (1.93‐fold) in muscle from *i*‐mn‐Sod1KO mice compared with control mice (Figure 4c).

### 3.5 Age‐associated decline in force generation is accelerated and exacerbated in *i*‐mn‐Sod1KO mice in older mice

**FIGURE 5:**
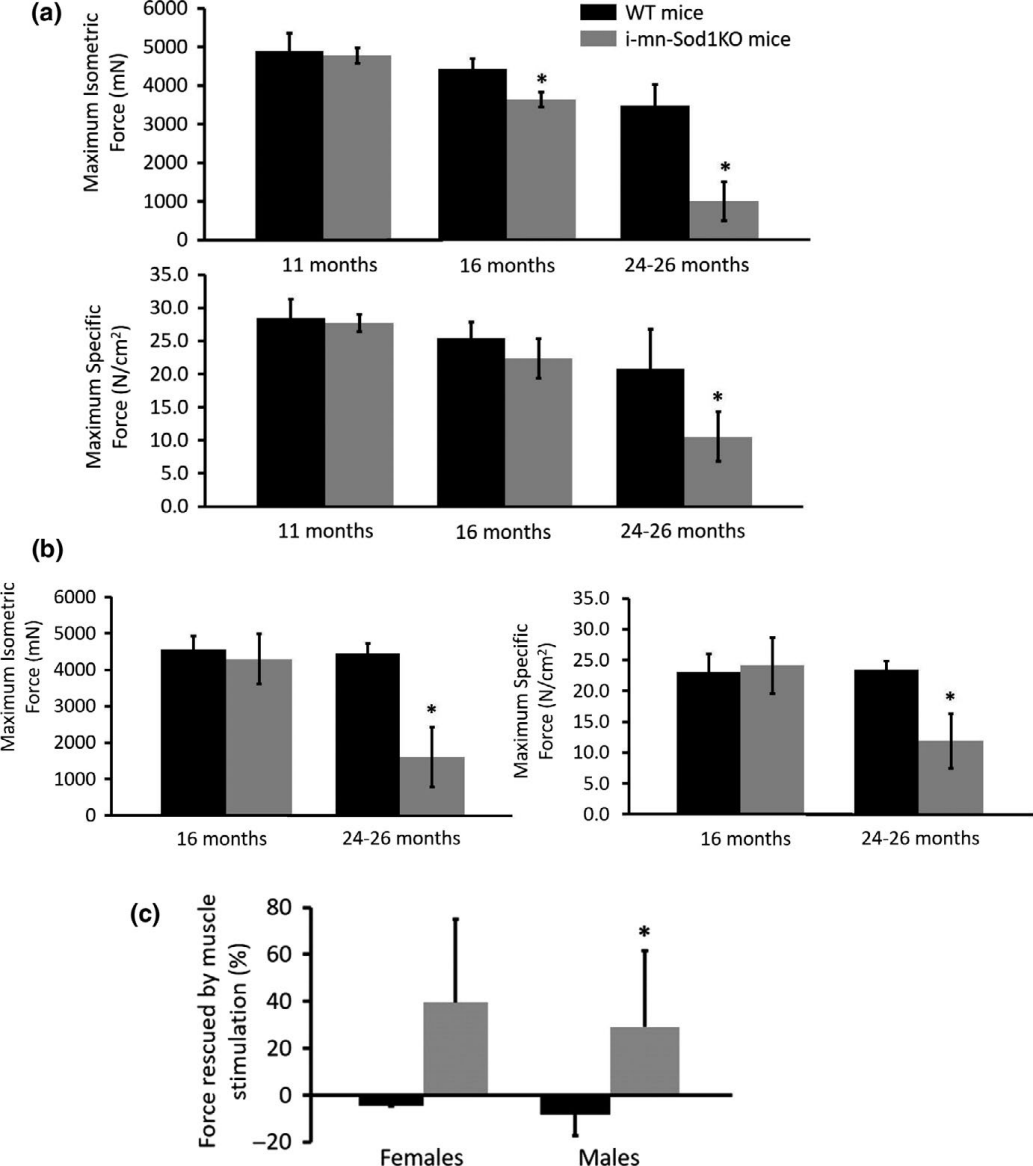
Force generation by gastrocnemius (GTN) muscles. Maximum isometric force expressed in milli‐newtons (mN) and maximum force normalized by total muscle fiber cross‐sectional area (N/cm^2^) for muscles of (a) female and (b) male mice with muscles activated by stimulation of the nerve. Deficits in absolute force generation for muscles of *i*‐mn‐Sod1KO compared with wild‐type (WT) mice were observed in female mice by 16 months that were explained by loss of muscle mass (i.e., no deficit in specific force). Both female and male mice showed deficits in specific force by 24 months of age. (c) The magnitude of the increase in force (% of values shown in panels a and b) when muscles of 24‐ to 26‐month‐old mice were activated by direct muscle stimulation. These data indicate that a portion of the specific force deficit in *i*‐mn‐Sod1KO mice is due to the presence of functionally denervated muscle fibers with NMJs that do not transmit an action potential. Data are expressed as mean ± *SD* and *significant difference from WT values at the same age

Based on the observation that compared with muscles of control mice, muscles of *i*‐mn‐Sod1KO mice show increased NMJ disruption, we tested whether there was an effect on force generation in response to nerve stimulation. Consistent with smaller masses of GTN muscles in *i*‐mn‐Sod1KO compared with control mice, maximum isometric force (*P*_o_) is significantly lower for muscles of i‐mn‐Sod1KO mice compared with wild‐type tamoxifen injected control values by 16 months in female mice (Figure 5a) and slightly later (by 24 months) in male mice (Figure 5b). The lower *P*_o_ at 16 months in female mice is not associated with a decrease in specific force indicating that the lower force is due entirely to muscle atrophy with no difference in the quality of the muscle in terms of force‐generating capacity. By 24 months, muscles of *i*‐mn‐Sod1KO mice displayed lower specific forces compared with wild‐type mice for both female and male mice (Figure 5a,b). Based on the observation that 65% of the NMJs in GTN muscles of *i*‐mn‐Sod1KO mice appeared either partially or fully denervated at 24 months (Figure 3b), we hypothesized that at least a portion of the weakness of muscles in *i*‐mn‐Sod1KO mice we observed at that age in response to activation through the nerve was due to functionally denervated NMJs that did not transmit an action potential to the muscle to generate contraction. To test this hypothesis, we tested whether any of the weakness could be rescued when the muscle was activated by direct muscle stimulation, bypassing the requirement for a functional NMJ. No differences were observed for wild‐type mice in maximum force generation using either nerve stimulation or direct muscle stimulation indicating that the NMJs in wild‐type muscles were largely functionally intact and capable of transmitting an action potential (Figure 5c). In contrast, force was increased ~30% in *i*‐mn‐Sod1KO mice when the NMJ was bypassed and muscles were activated directly indicating that a substantial number of NMJs in *i*‐mn‐Sod1KO mice did not transmit a nerve action potential to the muscle fiber, although the muscle fibers could be activated directly to contract. These data show that reduction of CuZnSOD in neurons results in the development over time of functionally denervated NMJs.

### 3.6 Muscle mitochondrial function is not altered in *i*‐mn‐Sod1KO mice, even at later ages

**FIGURE 6:**
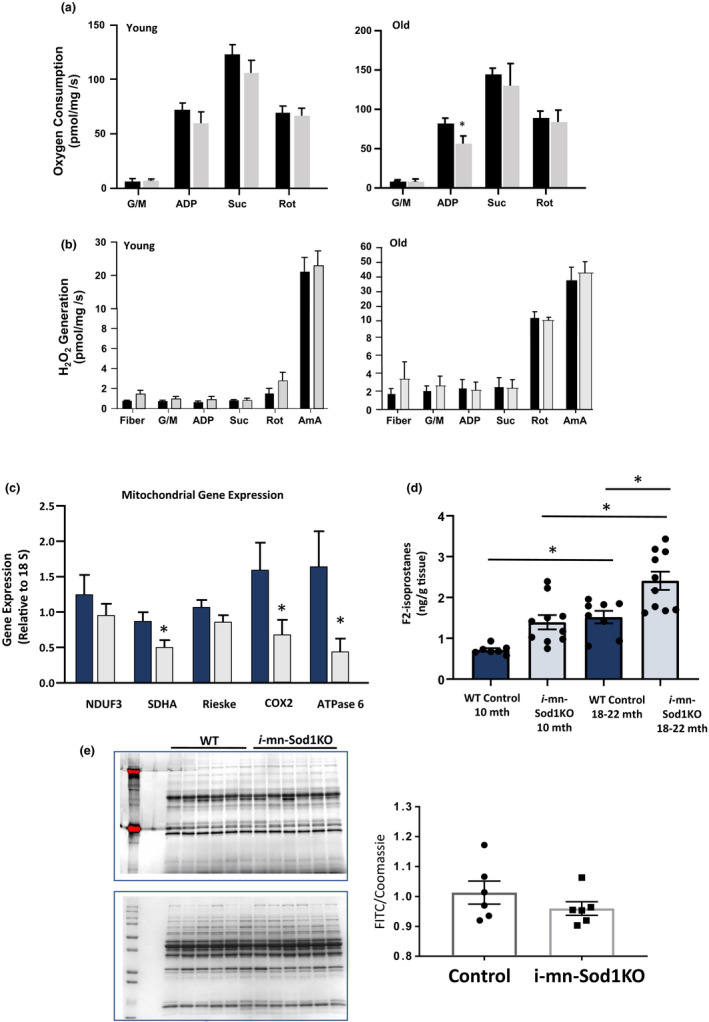
Mitochondrial function in permeabilized fibers from gastrocnemius muscles. (a) Mitochondrial oxygen consumption rates (OCR) and ROS generation were measured in permeabilized fibers from the red portion of the gastrocnemius muscle in female wild‐type and *i*‐mn‐Sod1KO young (9–11 months) and old (24–28 months) mice (*n* = 3–7/group). Substrates and inhibitors for mitochondria were sequentially added: glutamate (10 mM), malate (2 mM), ADP (5 mM), succinate (10 mM), rotenone (1 µM), Antimycin A (1 µM), and TMPD (0.5 mM) immediately followed by ascorbate (5 mM). Values are shown as means ± *SEM*. Abbreviations: AmA, Antimycin A; G/M, glutamate and malate; Rot, rotenone; Suc, succinate; TMPD, N,N,N′,N′‐tetramethyl‐p‐phenylenediamine. Data were analyzed by one way ANOVA. No statistically significant differences were found. (c) Relative transcript levels of mitochondrial complex proteins in gastrocnemius muscle in 24‐ to 26‐month‐old control and *i*‐mn‐Sod1KO mice (*n* = 5) determined by real‐time PCR. *Significant difference using Student's *t* test (*p* < .05) (d) F_2_‐isoprostane levels were measured in quadriceps muscle as a marker of oxidative stress. Isoprostanes are higher in both young and old *i*‐mn‐Sod1KO mice while mitochondrial function is not reduced. Data are expressed as mean ± *SEM* and represent *n* = 7 and *n* = 8 for young and old wild‐type respectively and *n* = 10 per group for young and old *i*‐mn‐Sod1KOmice. (e) Protein carbonyls in gastrocnemius muscle from wild‐type and *i*‐mn‐Sod1KO mice (*n* = 6). Top panel is FITC and bottom pannel Coomassie blue stained gel.

We have previously shown that peroxide generation from mitochondria is strongly correlated to denervation‐induced muscle atrophy (Muller et al., 2007). Here, we measured mitochondrial oxygen consumption and peroxide generation simultaneously using permeabilized muscle fibers from GTN muscles. Mitochondrial respiration and rates of hydrogen peroxide generation are not different in muscle from control and i‐mn‐Sod1KO mice, even in fibers isolated from 24‐ to 28‐month‐old mice (Figure 6a,b). To confirm that the mitochondrial number did not vary significantly between muscle fibers from control and *i*‐mn‐Sod1KO mice, we measured expression of VDAC, an outer mitochondrial membrane protein by Western blot analysis. The data in Figure S3 show similar levels of VDAC expression, supporting a similar mitochondrial content between the two groups. We also measured expression of a subset of genes representative of subunits present in electron transport chain Complexes I–V in GTN muscle from the older mice. Several of the genes show lower mRNA expression in the muscle from 24‐ to 26‐month‐old *i*‐mn‐Sod1KO mice (Figure 6C) suggesting a possible impact on mitochondria despite the lack of a measureable reduction in function.

### 3.7 Oxidative damage

F_2_‐Isoprostanes are a sensitive and reliable marker of oxidative stress. We measured the F_2_‐isoprostanes in quadriceps muscle from control and *i*‐mn‐Sod1KO mice (Figure 6d). The level of F_2_‐isoprostanes is increased by 94% in muscle tissue from the 10‐month‐old i‐mn‐Sod1KO mice compared with age‐matched control mice (NS, *p* < .06)) and by 111% in the 18‐ to 22‐month‐old mice compared with young control mice (*p* < .05). In the 18‐ to 22‐month‐old group, the *i*‐mn‐Sod1KO mice have a 59% increase in F_2_‐isoprostanes. Surprisingly however, we did not find an increase in protein carbonylation in the 18‐ to 22‐month‐old *i*‐mn‐Sod1KO mice (Figure 6d).

## 4 DISCUSSION

The major finding of this study is that reduced expression of the superoxide scavenger CuZnSOD in neuronal tissue, initiated postdevelopment in adult mice, results in an early loss of alpha motor neurons in the ventral spinal cord, followed by a later disruption of neuromuscular junctions and muscle atrophy and weakness in the older mice. Thus, while the reduction in CuZnSOD protein expression induced after development (at 2–4 months of age) restricted to neurons does indeed eventually lead to increased muscle atrophy and weakness, this effect is not evident until the mice are between 16 and 22 months of age (more than 1 year after deletion of the gene with tamoxifen). The delay in the presentation of muscle atrophy is observed despite alpha motor neuron loss being evident within a few months after *Sod1* deletion. These data support a critical role for innervation in maintaining muscle mass and strength with age but also indicate a robust ability of the neuromuscular system to exploit compensatory mechanisms to delay the development of the sarcopenia phenotype. The findings further suggest that muscle loss and weakness manifest only once the compensatory mechanisms are exhausted or after the onset of additional intrinsic aging effects in the neurons or muscle tissue. This “two hit” hypothesis suggests that loss of motor neurons will initiate muscle degeneration, but that the critical threshold for NMJ disruption and atrophy requires a “second hit”. While our experiments implicate motor neuron death as the first hit, the substantial delay between a measureable loss of motor neurons from the spinal cord and its manifestation as muscle atrophy indicates that there are compensatory mechanisms that protect the muscle from denervation‐induced fiber loss. The nature of the compensatory mechanisms are not clear, but it is reasonable to hypothesize that failure of one or more of these compensatory mechanisms represents the second hit. The compensatory mechanisms clearly must facilitate motor unit remodeling, that is, axonal branching from innervated motor units that rescue fibers in denervated motor units. Thus, protective compensatory mechanisms likely include factors that contribute to the maintenance of the ability of axonal sprouts to form, grow and be guided to denervated myofibers as well as facilitating the receptiveness of denervated myofibers to form and maintain stable “new” NMJs. Other experiments from our group show that restoration of postsynaptic cellular functions in *Sod1KO* mice, such as calcium handling by the myofibers and preservation of mitochondrial function, is also protective of muscle mass and function and thus could be argued as the second hit. Finally, we and others have reported that adaptive responses in muscle following denervation eventually fail (Chipman, Schachner, & Rafuse, 2014; Scalabrin et al., 2019; Vasilaki et al., 2010). Thus, the second hit may involve the muscle reaching a threshold past which it no longer maintains NMJ structure and function allowing downstream effects of muscle mass and function to occur. Alternatively, the remaining motor neurons may reach a threshold past which they cannot maintain increasing motor unit size resulting in further denervation and atrophy. Of course, some combination of these two possibilities could also collectively provide the second hit.

In an earlier study, we reported that reduction of CuZnSOD in Sod1 floxed mice using Cre recombinase expression driven by the promoter for nestin (n‐Sod1KO mice) resulted in only a very mild NMJ phenotype, a very slight reduction in force generation and no atrophy in the gastrocnemius muscle even at 20 months of age (Sataranatarajan et al., 2015). These results were intriguing and suggested that a phenotype was potentially developing but not complete in these mice. In retrospect, we considered whether the results were directly related to our choice of Cre recombinase model. The nestin‐Cre model is a constitutively expressed Cre recombinase that would direct deletion of *Sod1* beginning early in embryonic development and persist throughout the lifespan. The nestin protein is an intermediate filament protein and component of the cytoskeleton in neural stem cells that is also known to be expressed in a number of non‐neuronal tissues, including muscle satellite stem cells (Bernal & Arranz, 2018). Thus, the induction of *Sod1* deletion in this model would not only have been initiated early in development but would potentially have resulted in *Sod1* deletion in other tissues as well. For these reasons, in the current study we used an alternative Cre model, a motor neuron targeted Cre recombinase (Slick H Cre) and initiated the deletion in young adult mice. Although the extent of deletion we detected in the brain, spinal cord, and sciatic nerve in the *i*‐mn‐Sod1KO mice was slightly less than the deletion we previously achieved in the nestin‐Cre *Sod1* deletion model, the deletion of *Sod1* in the motor neurons in the Slick H Cre model appears to be very good, as demonstrated by our staining of motor neurons in the ventral spinal cord. Moreover, one would not anticipate more pronounced evidence of whole tissue deletion based on the heterogeneity of cell types present in these tissues. The deletion of *Sod1* targeted to neurons is a major advantage of this model and allows us to more definitely test the role of neuron versus muscle in the initiation and development of muscle atrophy and weakness.

To verify the response to the Slick H Cre recombinase, we measured motor neuron counts in the ventral horn of the lumbar spinal cord and found a significant loss of motor neuron number, decreased motor neuron area, and clasping behavior in response to tail suspension in the *i*‐mn‐Sod1KO mice compared with control mice, all phenotypes consistent with neuronal degenerative processes. By 10 months of age (i.e., 6–8 months after *Sod1* gene deletion), the *i*‐mn‐Sod1KO mice showed a 28% loss in alpha motor neurons compared with age‐matched wild‐type mice that was even greater (over 50%) in the older *i*‐mn‐Sod1KO mice. A significant age‐related loss of motor neurons was also evident by 18 to 22 months in control mice. Consistent with these data, we previously measured motor neuron counts in young (3‐ to 6‐month‐old) versus old wild‐type (24 to 27 month) C57Bl6 J mice and reported a similar (41%) decline in motor neuron number with age (Piekarz et al., 2020). These findings support a role for oxidative stress‐induced neuron loss in the spinal cord of the young *i*‐mn‐Sod1KO mice that is exacerbated as a function of age.

CuZnSOD protein is very high in neuronal tissue (0.5% of total protein), and it likely is critical for maintaining redox balance in neurons (Troy & Shelanski, 1994). Mice lacking CuZnSOD are known to exhibit progressive motor axonopathy and severe mitochondrial oxidation in neurons (Fischer, Li, Asress, Jones, & Glass, 2012). In addition, motor neurons are very sensitive to oxidative stress‐induced pathology (Gilgun‐Sherki, Melamed, & Offen, 2001) and increased oxidative stress in neurons is a contributing factor for pathogenesis of several neurological disease such as Alzheimer's disease, Parkinson disease, Huntington's disease, and amyotrophic lateral sclerosis (Kim, Kim, Rhie, & Yoon, 2015; Paloczi, Varga, Hasko, & Pacher, 2018; Shaw & Eggett, 2000). It has also been reported that CuZnSOD protein expression is high in motor neurons compared with other neurons, suggesting the importance of CuZnSOD in motor neurons (Bergeron, Petrunka, & Weyer, 1996). Importantly, *Sod1* deficient neurons are more susceptible to cell death due to axotomy (Reaume et al., 1996) ischemia (Kondo et al., 1997), and glutamate excitotoxicity (Schwartz, Reaume, Scott, & Coyle, 1998). Consistent with the impact of oxidative stress in motor neurons, conditional motor neuron deletion of glutathione peroxide 4 (GPX4), another antioxidant enzyme that functions to remove lipid peroxide‐induced damage, also leads to severe atrophy and paralysis (Chen et al., 2015). Finally, in a previous study we showed that neuron‐specific expression of *Sod1* in whole‐body knockout mice for CuZnSOD (*Sod1* ^−/−^ mice) prevented the accelerated muscle atrophy and NMJ disruption phenotype in these mice, and improved muscle function (Sakellariou et al., 2014). It is interesting to note here that our data from that study indicated that approximately 20% of the endogenous mouse CuZnSOD protein had been replaced with human CuZnSOD, suggesting that even a small amount of CuZnSOD is sufficient to protect motor neurons and prevent atrophy. In the current study, the later development of the phenotype of NMJ disruption, atrophy, and weakness suggests that we have deletion of *Sod1* from most, but perhaps not all alpha motor neurons. It is feasible that this residual CuZnSOD activity contributes to the mitigation of the phenotype in middle age and delay in NMJ disruption, atrophy, and weakness until later in the lifespan. Together these findings, all support the fact that elevated oxidative stress in motor neurons is highly correlated to muscle atrophy and degeneration, although the sequence of events leading from neuronal stress to NMJ disruption and atrophy and weakness remain to be clearly defined.

Loss of innervation in skeletal muscle could occur through changes in the neurons that lead to retraction of neurons from the acetylcholine receptor or changes in the postsynaptic components of the neuromuscular junction, the ultimate connection between neuronal and muscle tissue. Disruption of NMJ structure and function is a hallmark of aging in humans and rodent models and is associated with sarcopenia (Jackson & McArdle, 2016; Jang et al., 2012; McArdle, Pollock, Staunton, & Jackson, 2019; Valdez et al., 2010; Vasilaki et al., 2016, 2017) and several neurological disorders including ALS (Gould et al., 2006; Valdez, Tapia, Lichtman, Fox, & Sanes, 2012). We show here that targeted deletion of *Sod1* in motor neurons results in nearly 70% of junctions showing complete or partial denervation by 24 months of age, although only 22% are fully denervated. This is far less than the extent of denervation we have previously reported in the *Sod1* ^−/−^ mice where by 18–20 months of age 80% of the synapses are denervated (Jang et al., 2010). An additional point that should be considered is that we do not completely understand the relationship between the morphology of the NMJ and its functional capacity. It is possible that some percentage of the NMJs that appear normal are not completely functional or vice versa.

Our previous studies also show a clear connection between loss of innervation and increased generation of peroxides from mitochondria that could contribute to the downstream initiation and progression of muscle degeneration and atrophy as well as changes in force generation. We show here that the *i*‐mn‐Sod1KO mice do not show changes in oxygen consumption or mitochondrial peroxide generation even in the older mice, suggesting the loss of innervation is not extensive enough to cause peroxide generation in the *i*‐mn‐Sod1KO mice. We do however see some evidence of mitochondrial stress in muscle of the old *i*‐mn‐Sod1KO mice as shown in the reduced level of electron transport chain complexes. It is possible that the lack of mitochondrial phenotype is related to a compensation by the remaining fibers that do not drop out in response to loss of NMJS. This has been shown for example, in a mouse model of Amyotrophic Lateral Sclerosis (ALS), where there is a dramatic loss of NMJs and a preferential loss of Type IIB fast fibers while the remaining fibers become stronger and more fatigue resistant (Hegedus, Putman, Tyreman, & Gordon, 2008). On the other hand, it is possible that atrophy is due largely to fiber loss and the fibers that remain have sufficiently intact NMJs to maintain postsynaptic functions.

We measured F_2_‐isoprostanes as a measure of oxidative damage that is often associated with muscle atrophy in aging. Despite the lack of mitochondrial peroxide generation in the muscle from the *i*‐mn‐Sod1KO mice, F_2_‐isoprostane levels are increased in both the young and older cohorts of *i*‐mn‐Sod1ko mice and correlate roughly with extent of loss of muscle mass. This result suggests the absence of CuZnSOD in motor neurons leads to increased oxidative stress in skeletal muscle that is not generated by the mitochondria.

In conclusion, our findings show that oxidative stress induced by deletion of CuZnSOD can effectively initiate loss of motor neurons within a fairly short time frame after deletion that progresses with age. In contrast, the downstream phenotypes associated with sarcopenia, that is, NMJ disruption, muscle atrophy, and weakness, all appear later, with a significant delay after motor neuron deletion of CuZnSOD. It takes several months before the deletion of CuZnSOD from motor neurons leads to NMJ disruption, suggesting there is a threshold reached, past which the muscle and neuron cannot maintain intact NMJs. Muscle atrophy and weakness also appear on this delayed time frame, again supporting a threshold effect or requirement for a second hit in addition to motor neuron deficits. In addition, we expect that the surviving motor neurons expand their axonal arbors based on previous studies in nerve injury, partial denervation, and in aging where significant expansion of motor units occurs. These findings support an important role for motor neurons in initiating muscle degeneration but implicate the possibility of a requirement for an accumulation of damage postsynaptically before the full phenotype develops. Our future studies will seek to define the critical factors that contribute to the threshold effect.

## CONFLICT OF INTEREST

The authors declare that they have no conflict of interest.

## AUTHOR CONTRIBUTIONS

S.B. coordinated data collection for characterization of the mouse model, analyzed data, prepared figures, and drafted portions of the manuscript; N.P., C.A.S., and P.M. provided neuromuscular junction analysis; S.V.B. and P.M. conducted muscle function analysis; B.A and J.B. performed muscle mitochondrial analysis; K.P analyzed spinal cord sections for motor neuron counts and area and IHC analysis; R.Q, assisted S.B with experiment organization and sacrifice; A.V., A.R., A.M., M.J., S.V.B., and H.V.R. designed the experiments, wrote, and edited manuscript.

## Supporting information

Fig S1–S4

## Data Availability

The data that support the findings of this study are available from the corresponding author upon reasonable request.

## References

[acel13225-bib-0001] Ahn, B. , Ranjit, R. , Premkumar, P. , Pharaoh, G. , Piekarz, K. M. , Matsuzaki, S. , … Van Remmen, H. (2019). Mitochondrial oxidative stress impairs contractile function but paradoxically increases muscle mass via fibre branching. Journal of Cachexia, Sarcopenia and Muscle, 10(2), 411–428. 10.1002/jcsm.12375 PMC6463475 30706998

[acel13225-bib-0002] Barker, T. , & Traber, M. G. (2007). From animals to humans: Evidence linking oxidative stress as a causative factor in muscle atrophy. Journal of Physiology, 583(Pt 2), 421–422. 10.1113/jphysiol.2007.139378 17640928 PMC2277042

[acel13225-bib-0003] Bergeron, C. , Petrunka, C. , & Weyer, L. (1996). Copper/zinc superoxide dismutase expression in the human central nervous system. Correlation with selective neuronal vulnerability. American Journal of Pathology, 148(1), 273–279. 8546216 PMC1861609

[acel13225-bib-0004] Bernal, A. , & Arranz, L. (2018). Nestin‐expressing progenitor cells: function, identity and therapeutic implications. Cellular and Molecular Life Sciences, 75(12), 2177–2195. 10.1007/s00018-018-2794-z 29541793 PMC5948302

[acel13225-bib-0005] Bhaskaran, S. , Pharaoh, G. , Ranjit, R. , Murphy, A. , Matsuzaki, S. , Nair, B. C. , … Deepa, S. S. (2018). Loss of mitochondrial protease ClpP protects mice from diet‐induced obesity and insulin resistance. EMBO Reports, 19(3), 10.15252/embr.201745009 PMC5836096 29420235

[acel13225-bib-0006] Brooks, S. V. , & Faulkner, J. A. (1988). Contractile properties of skeletal muscles from young, adult and aged mice. Journal of Physiology, 404, 71–82. 10.1113/jphysiol.1988.sp017279 3253447 PMC1190815

[acel13225-bib-0007] Brooks, S. V. , & Faulkner, J. A. (1991). Maximum and sustained power of extensor digitorum longus muscles from young, adult, and old mice. Journal of Gerontology, 46(1), B28–B33. 10.1093/geronj/46.1.b28 1824709

[acel13225-bib-0008] Brooks, S. V. , & Faulkner, J. A. (1994). Isometric, shortening, and lengthening contractions of muscle fiber segments from adult and old mice. American Journal of Physiology, 267(2 Pt 1), C507–C513. 10.1152/ajpcell.1994.267.2.C507 8074185

[acel13225-bib-0009] Broome, C. S. , Kayani, A. C. , Palomero, J. , Dillmann, W. H. , Mestril, R. , Jackson, M. J. , … Mcardle, A. (2006). Effect of lifelong overexpression of HSP70 in skeletal muscle on age‐related oxidative stress and adaptation after nondamaging contractile activity. The FASEB Journal, 20(9), 1549–1551. 10.1096/fj.05-4935fje 16723383

[acel13225-bib-0010] Chen, L. , Hambright, W. S. , Na, R. , & Ran, Q. (2015). Ablation of the ferroptosis inhibitor glutathione peroxidase 4 in neurons results in rapid motor neuron degeneration and paralysis. Journal of Biological Chemistry, 290(47), 28097–28106. 10.1074/jbc.M115.680090 26400084 PMC4653669

[acel13225-bib-0011] Chipman, P. H. , Schachner, M. , & Rafuse, V. F. (2014). Presynaptic NCAM is required for motor neurons to functionally expand their peripheral field of innervation in partially denervated muscles. Journal of Neuroscience, 34(32), 10497–10510. 10.1523/JNEUROSCI.0697-14.2014 25100585 PMC4122797

[acel13225-bib-0012] Deepa, S. S. , Van Remmen, H. , Brooks, S. V. , Faulkner, J. A. , Larkin, L. , McArdle, A. , … Richardson, A. (2019). Accelerated sarcopenia in Cu/Zn superoxide dismutase knockout mice. Free Radical Biology and Medicine, 132, 19–23. 10.1016/j.freeradbiomed.2018.06.032 30670156 PMC6405207

[acel13225-bib-0013] Fischer, L. R. , Li, Y. J. , Asress, S. A. , Jones, D. P. , & Glass, J. D. (2012). Absence of SOD1 leads to oxidative stress in peripheral nerve and causes a progressive distal motor axonopathy. Experimental Neurology, 233(1), 163–171. 10.1016/j.expneurol.2011.09.020 21963651 PMC4068963

[acel13225-bib-0014] Gilgun‐Sherki, Y. , Melamed, E. , & Offen, D. (2001). Oxidative stress induced‐neurodegenerative diseases: The need for antioxidants that penetrate the blood brain barrier. Neuropharmacology, 40(8), 959–975. 10.1016/S0028-3908(01)00019-3 11406187

[acel13225-bib-0015] Gould, T. W. , Buss, R. R. , Vinsant, S. , Prevette, D. , Sun, W. , Knudson, C. M. , … Oppenheim, R. W. (2006). Complete dissociation of motor neuron death from motor dysfunction by Bax deletion in a mouse model of ALS. Journal of Neuroscience, 26(34), 8774–8786. 10.1523/Jneurosci.2315-06.2006 16928866 PMC6674380

[acel13225-bib-0016] Gusel'nikova, V. V. , & Korzhevskiy, D. E. (2015). NeuN as a neuronal nuclear antigen and neuron differentiation marker. Acta Naturae, 7(2), 42–47. 26085943 PMC4463411

[acel13225-bib-0017] Hegedus, J. , Putman, C. T. , Tyreman, N. , & Gordon, T. (2008). Preferential motor unit loss in the SOD1 G93A transgenic mouse model of amyotrophic lateral sclerosis. Journal of Physiology, 586(14), 3337–3351. 10.1113/jphysiol.2007.149286 18467368 PMC2538809

[acel13225-bib-0018] Jackson, M. J. , & McArdle, A. (2016). Role of reactive oxygen species in age‐related neuromuscular deficits. Journal of Physiology, 594(8), 1979–1988. 10.1113/JP270564 26870901 PMC4933106

[acel13225-bib-0019] Jang, Y. C. , Liu, Y. , Hayworth, C. R. , Bhattacharya, A. , Lustgarten, M. S. , Muller, F. L. , … Van Remmen, H. (2012). Dietary restriction attenuates age‐associated muscle atrophy by lowering oxidative stress in mice even in complete absence of CuZnSOD. Aging Cell, 11(5), 770–782. 10.1111/j.1474-9726.2012.00843.x 22672615 PMC3444532

[acel13225-bib-0020] Jang, Y. C. , Lustgarten, M. S. , Liu, Y. , Muller, F. L. , Bhattacharya, A. , Liang, H. , … Van Remmen, H. (2010). Increased superoxide in vivo accelerates age‐associated muscle atrophy through mitochondrial dysfunction and neuromuscular junction degeneration. FASEB Journal, 24(5), 1376–1390. 10.1096/fj.09-146308 20040516 PMC2987499

[acel13225-bib-0021] Kim, G. H. , Kim, J. E. , Rhie, S. J. , & Yoon, S. (2015). The role of oxidative stress in neurodegenerative diseases. Experimental Neurobiology, 24(4), 325–340. 10.5607/en.2015.24.4.325 26713080 PMC4688332

[acel13225-bib-0022] Kondo, T. , Reaume, A. G. , Huang, T.‐T. , Carlson, E. , Murakami, K. , Chen, S. F. , … Chan, P. H. (1997). Reduction of CuZn‐superoxide dismutase activity exacerbates neuronal cell injury and edema formation after transient focal cerebral ischemia. Journal of Neuroscience, 17(11), 4180–4189. 9151735 10.1523/JNEUROSCI.17-11-04180.1997 PMC6573543

[acel13225-bib-0023] Larkin, L. M. , Davis, C. S. , Sims‐Robinson, C. , Kostrominova, T. Y. , Remmen, H. V. , Richardson, A. , … Brooks, S. V. (2011). Skeletal muscle weakness due to deficiency of CuZn‐superoxide dismutase is associated with loss of functional innervation. American Journal of Physiology‐Regulatory Integrative and Comparative Physiology, 301(5), R1400–R1407. 10.1152/ajpregu.00093.2011 PMC3213934 21900648

[acel13225-bib-0024] Macpherson, P. C. D. , Farshi, P. , & Goldman, D. (2015). Dach2‐Hdac9 signaling regulates reinnervation of muscle endplates. Development, 142(23), 4038–4048. 10.1242/dev.125674 26483211 PMC4712835

[acel13225-bib-0025] McArdle, A. , Pollock, N. , Staunton, C. A. , & Jackson, M. J. (2019). Aberrant redox signalling and stress response in age‐related muscle decline: Role in inter‐ and intra‐cellular signalling. Free Radical Biology and Medicine, 132, 50–57. 10.1016/j.freeradbiomed.2018.11.038 30508577 PMC6709668

[acel13225-bib-0026] Muller, F. L. , Song, W. , Jang, Y. C. , Liu, Y. , Sabia, M. , Richardson, A. , & Van Remmen, H. (2007). Denervation‐induced skeletal muscle atrophy is associated with increased mitochondrial ROS production. American Journal of Physiology: Regulatory, Integrative and Comparative Physiology, 293(3), R1159–R1168. 10.1152/ajpregu.00767.2006 17584954

[acel13225-bib-0027] Muller, F. L. , Song, W. , Liu, Y. , Chaudhuri, A. , Pieke‐Dahl, S. , Strong, R. , … Van Remmen, H. (2006). Absence of CuZn superoxide dismutase leads to elevated oxidative stress and acceleration of age‐dependent skeletal muscle atrophy. Free Radical Biology and Medicine, 40(11), 1993–2004. 10.1016/j.freeradbiomed.2006.01.036 16716900

[acel13225-bib-0028] Paloczi, J. , Varga, Z. V. , Hasko, G. , & Pacher, P. (2018). Neuroprotection in oxidative stress‐related neurodegenerative diseases: Role of endocannabinoid system modulation. Antioxidants & Redox Signaling, 29(1), 75–108. 10.1089/ars.2017.7144 28497982 PMC5984569

[acel13225-bib-0029] Piekarz, K. M. , Bhaskaran, S. , Sataranatarajan, K. , Street, K. , Premkumar, P. , Saunders, D. , … Van Remmen, H. (2020). Molecular changes associated with spinal cord aging. Geroscience, 42(2), 765–784. 10.1007/s11357-020-00172-6 32144690 PMC7205981

[acel13225-bib-0030] Qaisar, R. , Bhaskaran, S. , Premkumar, P. , Ranjit, R. , Natarajan, K. S. , Ahn, B. , … Van Remmen, H. (2018). Oxidative stress‐induced dysregulation of excitation‐contraction coupling contributes to muscle weakness. Journal of Cachexia, Sarcopenia and Muscle, 9(5), 1003–1017. 10.1002/jcsm.12339 PMC6204588 30073804

[acel13225-bib-0031] Reaume, A. G. , Elliott, J. L. , Hoffman, E. K. , Kowall, N. W. , Ferrante, R. J. , Siwek, D. R. , … Snider, W. D. (1996). Motor neurons in Cu/Zn superoxide dismutase‐deficient mice develop normally but exhibit enhanced cell death after axonal injury. Nature Genetics, 13(1), 43–47. 10.1038/ng0596-43 8673102

[acel13225-bib-0032] Sakellariou, G. K. , Davis, C. S. , Shi, Y. , Ivannikov, M. V. , Zhang, Y. , Vasilaki, A. , … Brooks, S. V. (2014). Neuron‐specific expression of CuZnSOD prevents the loss of muscle mass and function that occurs in homozygous CuZnSOD‐knockout mice. FASEB Journal, 28(4), 1666–1681. 10.1096/fj.13-240390 24378874 PMC3963022

[acel13225-bib-0033] Sakellariou, G. K. , Pye, D. , Vasilaki, A. , Zibrik, L. , Palomero, J. , Kabayo, T. , … Jackson, M. J. (2011). Role of superoxide‐nitric oxide interactions in the accelerated age‐related loss of muscle mass in mice lacking Cu, Zn superoxide dismutase. Aging Cell, 10(5), 749–760. 10.1111/j.1474-9726.2011.00709.x 21443684 PMC3531889

[acel13225-bib-0034] Sataranatarajan, K. , Qaisar, R. , Davis, C. , Sakellariou, G. K. , Vasilaki, A. , Zhang, Y. , … Van Remmen, H. (2015). Neuron specific reduction in CuZnSOD is not sufficient to initiate a full sarcopenia phenotype. Redox Biology, 5, 140–148. 10.1016/j.redox.2015.04.005 25917273 PMC5022075

[acel13225-bib-0035] Scalabrin, M. , Pollock, N. , Staunton, C. A. , Brooks, S. V. , McArdle, A. , Jackson, M. J. , & Vasilaki, A. (2019). Redox responses in skeletal muscle following denervation. Redox Biology, 26, 101294 10.1016/j.redox.2019.101294 31450104 PMC6831873

[acel13225-bib-0036] Schwartz, P. J. , Reaume, A. , Scott, R. , & Coyle, J. T. (1998). Effects of over‐ and under‐expression of Cu, Zn‐superoxide dismutase on the toxicity of glutamate analogs in transgenic mouse striatum. Brain Research, 789(1), 32–39. 10.1016/s0006-8993(97)01469-8 9602043

[acel13225-bib-0037] Shaw, P. J. , & Eggett, C. J. (2000). Molecular factors underlying selective vulnerability of motor neurons to neurodegeneration in amyotrophic lateral sclerosis. Journal of Neurology, 247, 17–27. 10.1007/BF03161151 10795883

[acel13225-bib-0038] Snider, T. A. , Richardson, A. , Stoner, J. A. , & Deepa, S. S. (2018). The geropathology grading platform demonstrates that mice null for Cu/Zn‐superoxide dismutase show accelerated biological aging. Geroscience, 40(2), 97–103. 10.1007/s11357-018-0008-0 29478190 PMC5964058

[acel13225-bib-0039] Troy, C. M. , & Shelanski, M. L. (1994). Down‐regulation of copper/zinc superoxide dismutase causes apoptotic death in PC12 neuronal cells. Proceedings of the National Academy of Unites States of America, 91(14), 6384–6387. 10.1073/pnas.91.14.6384 PMC44206 8022792

[acel13225-bib-0040] Valdez, G. , Tapia, J. C. , Kang, H. , Clemenson, G. D. , Gage, F. H. , Lichtman, J. W. , & Sanes, J. R. (2010). Attenuation of age‐related changes in mouse neuromuscular synapses by caloric restriction and exercise. Proceedings of the National Academy of Sciences of the United States of America, 107(33), 14863–14868. 10.1073/pnas.1002220107 20679195 PMC2930485

[acel13225-bib-0041] Valdez, G. , Tapia, J. C. , Lichtman, J. W. , Fox, M. A. , & Sanes, J. R. (2012). Shared resistance to aging and ALS in neuromuscular junctions of specific muscles. PLoS One, 7(4), e34640 10.1371/journal.pone.0034640 22485182 PMC3317643

[acel13225-bib-0042] Vasilaki, A. , Pollock, N. , Giakoumaki, I. , Goljanek‐Whysall, K. , Sakellariou, G. K. , Pearson, T. , … McArdle, A. (2016). The effect of lengthening contractions on neuromuscular junction structure in adult and old mice. Age, 38(4), 259–272. 10.1007/s11357-016-9937-7 27470432 PMC5061675

[acel13225-bib-0043] Vasilaki, A. , Richardson, A. , Van Remmen, H. , Brooks, S. V. , Larkin, L. , McArdle, A. , & Jackson, M. J. (2017). Role of nerve‐muscle interactions and reactive oxygen species in regulation of muscle proteostasis with ageing. Journal of Physiology, 595(20), 6409–6415. 10.1113/Jp274336 28792061 PMC5638895

[acel13225-bib-0044] Vasilaki, A. , Simpson, D. , McArdle, F. , McLean, L. , Beynon, R. J. , Van Remmen, H. , … Jackson, M. J. (2007). Formation of 3‐nitrotyrosines in carbonic anhydrase III is a sensitive marker of oxidative stress in skeletal muscle. Proteomics Clinical Applications, 1(4), 362–372. 10.1002/prca.200600702 21136689

[acel13225-bib-0045] Vasilaki, A. , van der Meulen, J. H. , Larkin, L. , Harrison, D. C. , Pearson, T. , Van Remmen, H. , … McArdle, A. (2010). The age‐related failure of adaptive responses to contractile activity in skeletal muscle is mimicked in young mice by deletion of Cu, Zn superoxide dismutase. Aging Cell, 9(6), 979–990. 10.1111/j.1474-9726.2010.00635.x 20883524 PMC3437493

[acel13225-bib-0046] Young, P. , Qiu, L. , Wang, D. , Zhao, S. , Gross, J. , & Feng, G. (2008). Single‐neuron labeling with inducible Cre‐mediated knockout in transgenic mice. Nature Neuroscience, 11(6), 721–728. 10.1038/nn.2118 18454144 PMC3062628

[acel13225-bib-0047] Zhang, Y. , Davis, C. , Sakellariou, G. K. , Shi, Y. , Kayani, A. C. , Pulliam, D. , … Van Remmen, H. (2013). CuZnSOD gene deletion targeted to skeletal muscle leads to loss of contractile force but does not cause muscle atrophy in adult mice. FASEB Journal, 27(9), 3536–3548. 10.1096/fj.13-228130 23729587 PMC3752542

