# Supplementary material for: Neuron‐specific deletion of CuZnSOD leads to an advanced sarcopenic phenotype in older mice
Source: Aging Cell. 2020 Sep 4;19(10):e13225. doi: 10.1111/acel.13225 (PMC7576239; doi:10.1111/acel.13225)

Supplemental Figure 1. Muscle masses normalized for body mass for A) extensor digitorum longus (EDL), B) soleus (SOL), C) tibialis anterior (TA), and D) quadriceps (Quad) muscles of WT and i-mn-Sod1KO mice at 11 months (black bars), 16 months (gray bars), and 24 months (white bars). Data are presented as means ± S.D. Bars marked with the same letter are not significantly different from each other by two-factor ANOVA, with Tukey multiple comparisons post-hoc tests.

Supplemental Figure 2: Western blot analysis of RING-finger protein-1 (MURF1) in gastrocnemius muscle. A) Representative western blot of MURF1 in WT and i-mn-Sod1KO mice at 24 months of age. B) Graphical representation of MURF1 expression normalized to Ponceau stain. Bars represent mean± SEM for WT (n=6) and i-mn-Sod1KO (n=7).

Supplemental Figure 3. Western blot analysis of voltage dependent anion channel (VDAC) in gastrocnemius muscle. A) Representative western blot of VDAC in WT and i-mn-Sod1KO mice at 24 months of age. B) Graphical representation of VDAC expression normalized to GAPDH. Bars represent mean± SEM for WT (n=6) and i-mn-Sod1KO (n=7).

Supplemental Figure 4. CuZnSOD protein expression in spleen. A) Representative western blot of CuZnSOD in WT and i-mn-Sod1KO mice spleen at 24 months of age. B) Graphical representation of CuZnSOD expression normalized to GAPDH. Bars represent mean± SEM for WT (n=3) and i-mn-Sod1KO (n=3).

Supplemental Figure 1.

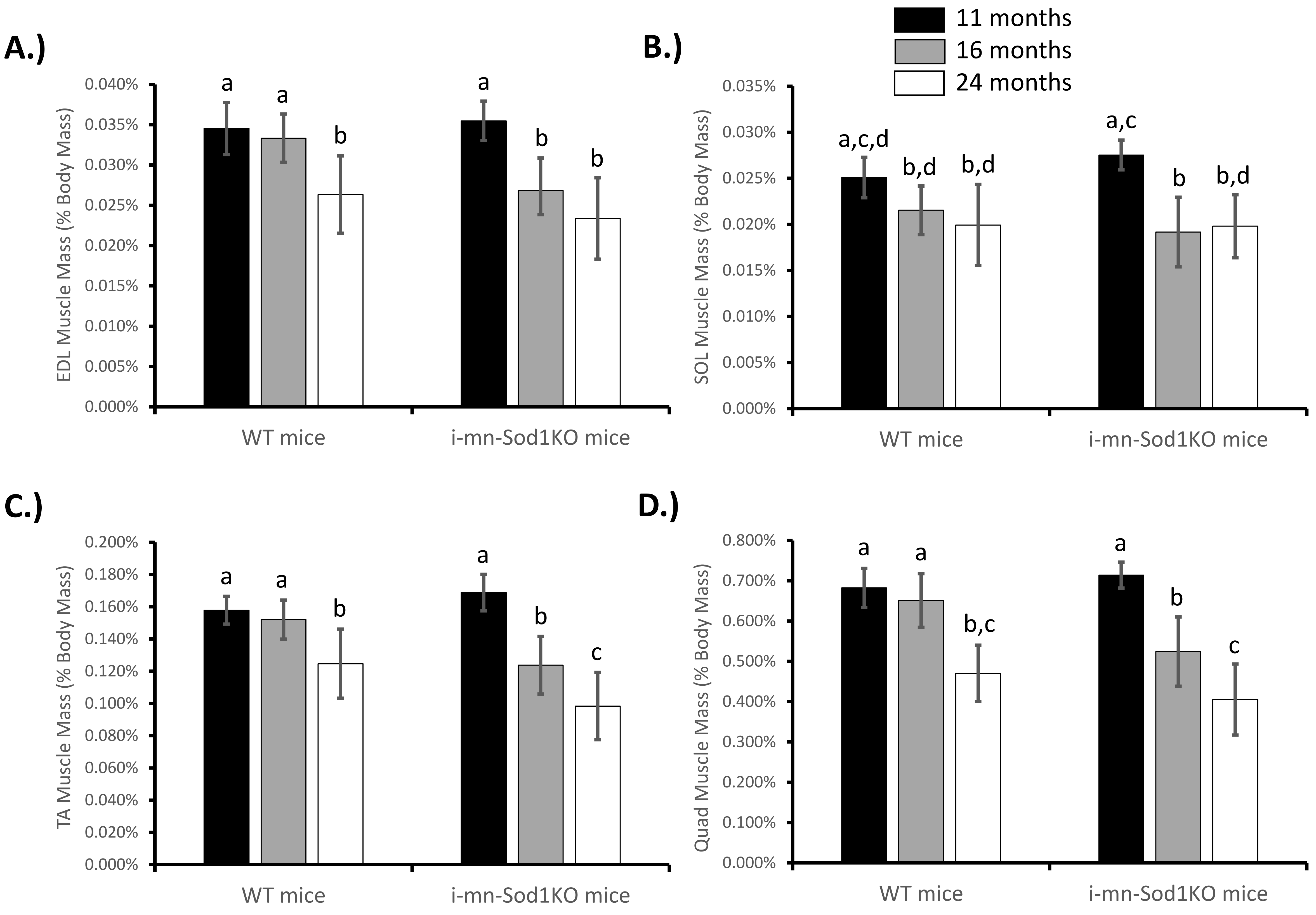

Supplemental Figure 2.

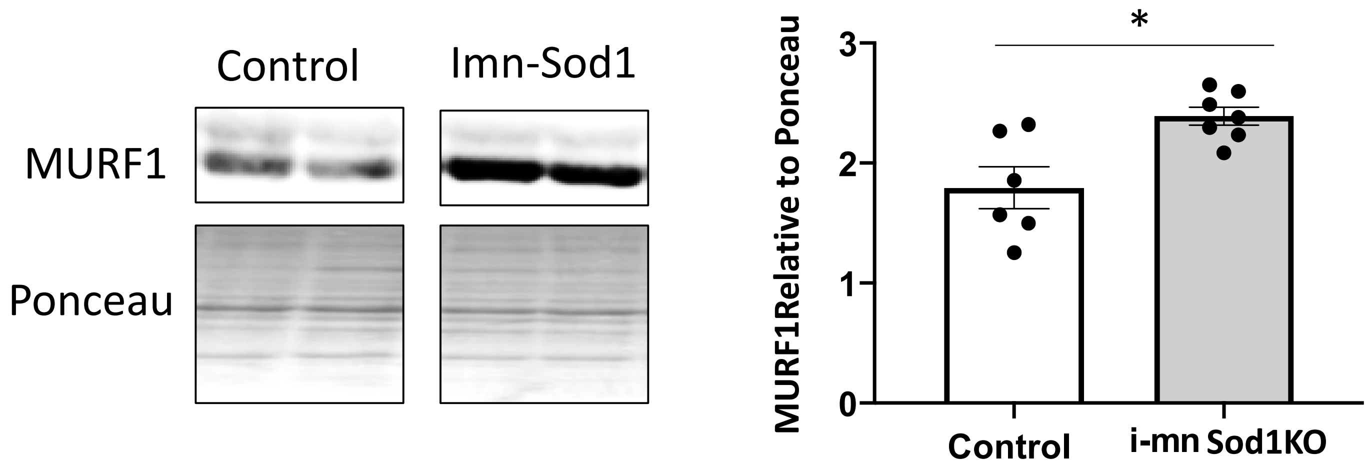

Supplemental Figure 3.

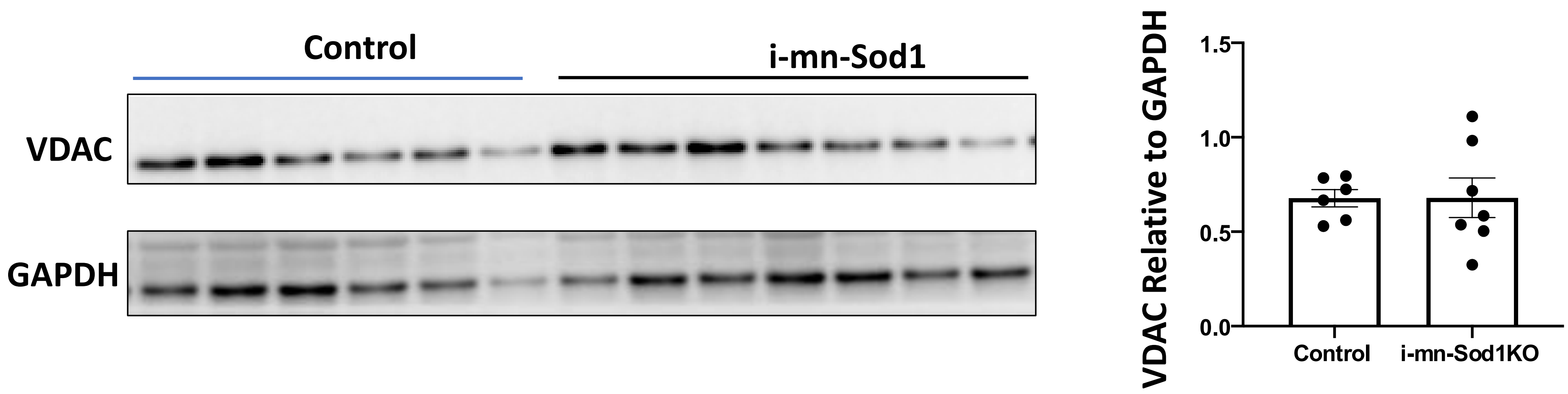

Supplemental Figure 4.

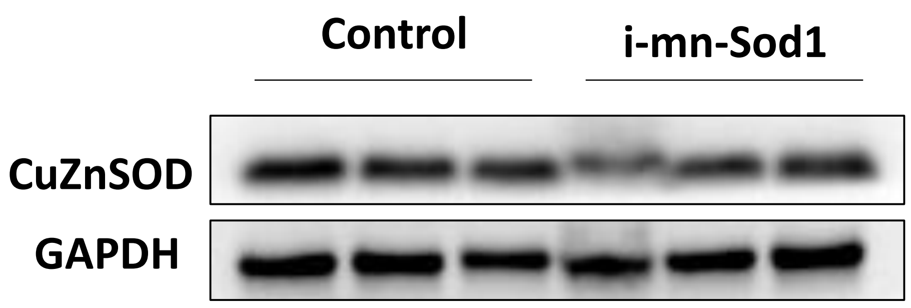

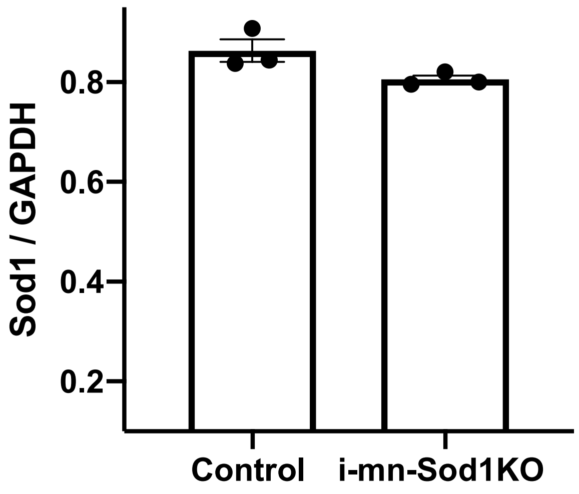

Supplement: Supplementary file 1 — Fig S1–S4 [file ACEL-19-e13225-s001.pdf]
